# Primary neoplasms of the parapharyngeal space: diagnostic and therapeutic pearls and pitfalls

**DOI:** 10.1007/s00405-021-06718-4

**Published:** 2021-03-19

**Authors:** Olcay Cem Bulut, Roland Giger, Ashwag Alwagdani, Nada Aldabal, Albrecht Stenzinger, Samuel Heimgartner, Lluís Nisa, Urs Borner

**Affiliations:** 1 Department of Otorhinolaryngology - Head and Neck Surgery, Inselspital, Bern University Hospital, Freiburgstrasse, 3010 Bern, Switzerland; 2 Department of Otorhinolaryngology - Head and Neck Surgery, SLK Kliniken Am Gesundbrunnen, 74078 Heilbronn, Germany; 3 Department of Pathology, University Hospital Heidelberg, 69120 Heidelberg, Germany

**Keywords:** Head and neck, Parapharyngeal space, Neoplasm, Tumor

## Abstract

**Purpose:**

Parapharyngeal space neoplasms (PSNs) are rare tumors of the head and neck region. In this study, we report our institutional experience with PSNs over a 27-years period.

**Methods:**

Patients treated between 1992 and 2018 were identified through our tumor board database. Data concerning demographics, clinical presentation, disease features, treatment, complications and follow-up were obtained retrospectively.

**Results:**

In total, 48 patients were identified. Most patients had benign tumors (67.5%), with pleomorphic adenoma and schwannoma being the most frequent entities. Malignant tumors represented the remaining 32.5% of neoplasms. Concerning tissue of origin, 67.5% of neoplasms originated from salivary glands and 17.5% were neurogenic. The vast majority of PSNs required open surgical approaches (77%). The most frequent reversible and irreversible complications included paralysis of facial, vagal, and hypoglossal nerves (transient 62.5%, permanent 31.3%). Tumor recurrences occurred in 16.7% of our patients.

**Conclusion:**

Neoplasms of the parapharyngeal space (PPS) are rare. In our series, consistent with the literature, most patients had benign tumors. Fine-needle aspiration cytology (FNAC) and/or transoral biopsy in selected cases combined with radiographic imaging are helpful to plan the optimal approach (open/transoral) and extent of primary surgery. Close follow-up in malignant neoplasms is crucial to assess recurrence early. We present one of the largest recent studies on PPS tumors treated in a center. Given the low incidence of these tumors, our results contribute to the existing sparse evidence regarding the management and outcome of such tumors.

## Introduction

Parapharyngeal space neoplasms (PSNs) are rare tumors, accounting only for approximately 0.5–1% of all head and neck neoplasms [1–4]. The parapharyngeal space (PPS) is a fascial space delimited by the hyoid bone and the skull base in the cranio-caudal axis and the buccopharyngeal fascia medially. It is bordered by the carotid sheath posterio-laterally and the retropharyngeal space posterio-medially. The fascia running posteriorly from the styloid process to the tensor veli palatine muscle divides the PPS into the pre- and the post-styloid compartments [1, 2, 4, 5].

Given the deep location of the tumors in a virtual space, PSNs may have a relatively long progression before becoming symptomatic; therefore, these tumors tend to achieve considerable volumes by the time of diagnosis [6]. There is some controversy regarding clinical classification of PSNs. In a systematic review, Riffat et al. reported that many authors included all deep lobe parotid gland tumors in PSNs [7]. However, not all neoplasms of the deep lobe of the parotid gland belong to PPS. Riffat et al. proposed that only lesions affecting at least the retromandibular part of the deep lobe of the parotid gland should be considered as PSNs [7].

PSNs classically present with medial displacement of the lateral oropharyngeal wall or the tonsil, a mandibular angle mass, pain, cranial nerve (CN) involvement (CN VII and IX to XII), and dysphagia [8]. The majority of all PSNs are benign, with a reported rate of malignant tumors ranging from 15 to 27% [1, 4, 5, 7, 9–11]. More than 40 different types of neoplasms can be found [7, 12, 13].

Tumors may arise primarily in the PPS, invade it by contiguity (e.g., tumors of the parotid gland, nasopharynx, oropharynx, or infratemporal fossa), or occur as distant metastases (e.g., cancer of the thyroid gland, other head and neck sites, or the kidney) [14–16]. Importantly, symptoms due to metastasization to the PPS can be the first manifestation of certain primaries [14–17]. Most primary PSNs are either salivary (35–45%) or neurogenic (35–41%); whereas, other histological types, such as hemangiomas, meningiomas, or lipomas are extremely rare [1, 2, 4, 5].

Treatment typically requires a surgical approach to the PPS. Surgical strategies for PSNs are challenging, given the deep location, the complex anatomy and the structures contained within this region [12].

The aim of this study is to report our institutional experience with primary PSNs over a 27-years period reviewing the pathology, surgical treatment, complications and tumor outcome.

## Methods

Approval of this study was granted by our institutional board review (*Direktion Lehre und Forschung, Inselspital – Universitätsspital Bern,* Switzerland).

Patients potentially suitable for inclusion in this study were identified through chart review. Inclusion criteria were: (a) histologically and radiologically proven primary PSNs; (b) availability of data, including basic demographics, diagnostic work-up findings (both radiological and histological), postoperative course, and clinical follow-up data, of at least 6 months for patients without events (recurrence, death); and (c) management period between January 1992 and December 2018. Patients with distant metastases to the PPS and with tumors that invaded the PPS by contiguity were excluded.

Three authors (OCB, UB and LN) performed chart review and extraction of data. Extracted data included patient demographics, clinical presentation, work-up, surgical treatment, histopathology, adjuvant therapy, complications and outcome. Data are presented as summary statistics.

## Results

### Patient and tumor features (Table 1)

**Table 1:** Summarized clinical and diagnostic aspects of PSNs (*n* = 48)

Features	No. of cases	%
Demographic		
* Male*	25	52.1
* Female*	23	47.9
Presentation		
* Neck swelling*	35	72.9
* Dysphagia*	19	39.6
* Pain*	16	33.3
* Snoring*	13	27.1
* Hoarseness*	8	16.7
* Asymptomatic*	7	14.6
Imaging		
* MRI*	46	95.8
* CT*	20	41.7
* PET/CT*	4	8.3
Diagnostic methods		
* US-guided FNAC*	29	60.4
* Transoral incisional biopsy*	11	22.9
* Both*	2	4.2
Histopathological findings		
* Benign tumor*	33	68.8
* Pleomorphic adenoma*	24	50
* Schwannoma*	6	12.5
* Cystadenolymphoma*	1	2.1
* Lipoma*	1	2.1
* Hemangioma*	1	2.1
***Malignant tumor***	15	31.2
* Carcinoma ex pleomorphic adenoma*	4	8.3
* Mucoepidermoid carcinoma*	2	4.2
* Rhabdomyosarcoma*	2	4.2
* Poorly differentiated salivary duct carcinoma*	2	4.2
* Liposarcoma*	1	2.1
* Malignant peripheral nerve sheath tumor*	1	2.1
* Adenocarcinoma*	1	2.1
* Undifferentiated sarcoma*	1	2.1
* Synovial sarcoma*	1	2.1

*CT* computed tomography, *FNAC* fine-needle aspiration cytology, *MRI* magnetic resonance imaging, *PET* positron emission tomography, *US* ultrasonography

Our search identified 48 patients that fulfilled the inclusion criteria. Mean age at diagnosis was 52.8 ± 18.7 years (4.2–88.3). Regarding gender, 25 (52.1%) patients were male and 23 (47.9%) were female.

While most patients presented some non-specific symptoms related to their PSNs (neck swelling, dysphagia and pain), 14.6% of the patients were asymptomatic and the diagnosis was mostly due to an accidental finding by imaging or dentist examination. The most common complaint at presentation was a swelling of the inferior part of the parotid region/the mandibular angle. The other reported symptoms are summarized in Table 1.

**Fig. 1:**
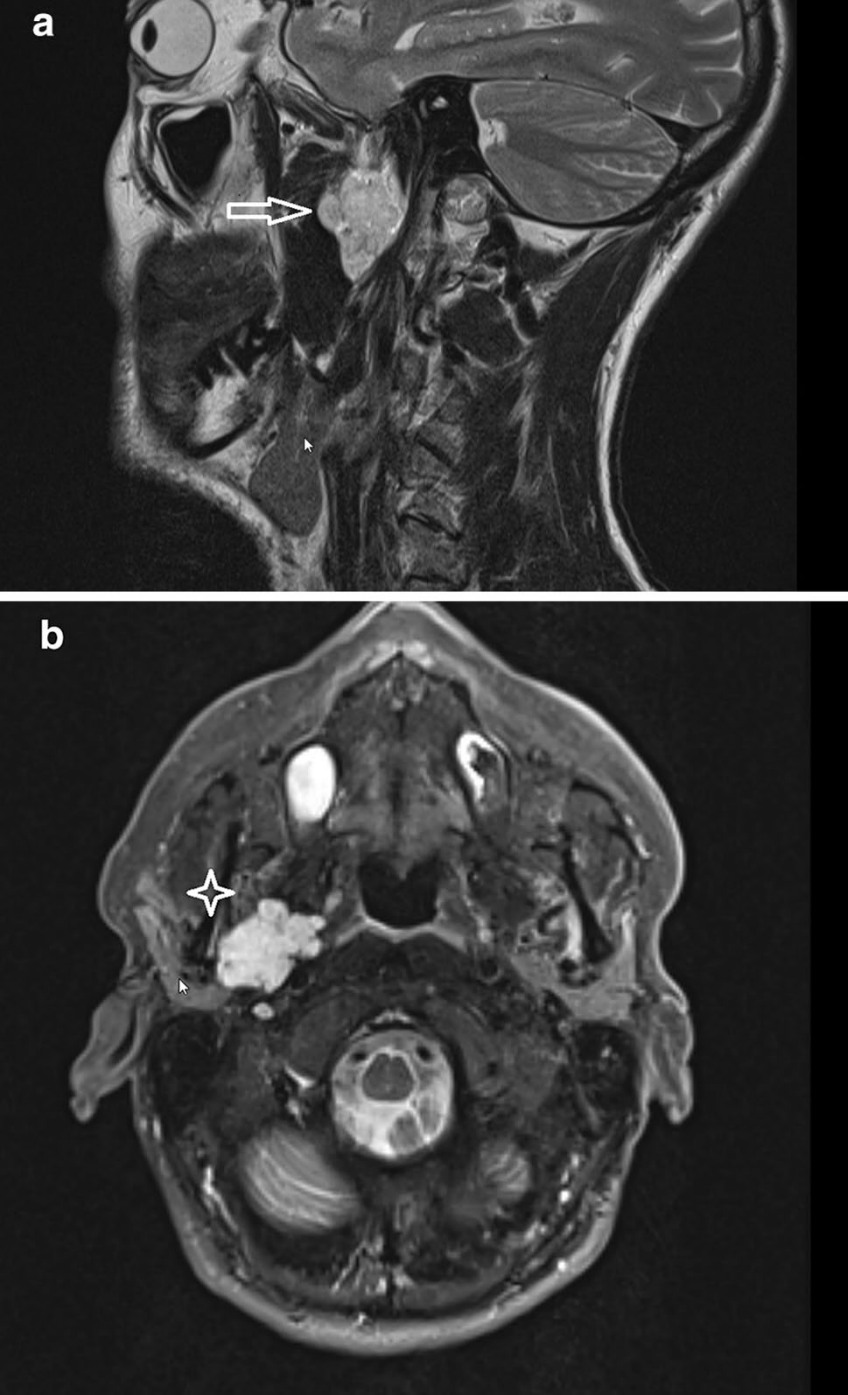
**a** Parasagittal MRI view of a parapharyngeal pleomorphic adenoma (arrow) (23 × 31 mm; T2). **b** Axial MRI view of a parapharyngeal pleomorphic adenoma (cross) (17 × 24 mm; T2)

Within the diagnostic work-up, all patients underwent at least one imaging exam, most frequently a MRI (Table 1). Average tumor size was 4.6 ± 1.3 cm. Figure 1a and b depicts a MRI from a patient with a parapharyngeal pleomorphic adenoma. Clinical and imaging work-up, and ruled out a vascular lesion, we attempted obtaining diagnosis by fine-needle aspiration cytology (FNAC) and/or transoral incisional biopsy in cases where a transoral resection was intended. We performed ultrasound (US)-guided FNAC in 29 cases (60.4%), and 11 cases (22.9%) underwent transoral incisional biopsy. Two patients underwent both. In terms of diagnostic accuracy, FNAC was not conclusive in ten cases (34.5%). Out of the 19 remaining cases in which FNAC yielded a diagnosis of neoplasia, we found a sensitivity of 50%, specificity of 100%, positive predictive value of 100% and negative predictive value of 88.2% to detect malignancy, with an accuracy of 89.5%. Regarding specific histological subtypes, FNAC correctly diagnosed 13/15 pleomorphic adenomas. As for transoral incisional biopsies, one case was not conclusive (the final diagnosis was carcinoma ex pleomorphic adenoma). All remaining cases properly allowed identifying the benign/malignant nature of PSNs, while only one case of undifferentiated sarcoma was initially stated as possible mucoepidermoid carcinoma.

Definitive histopathology of the resected tumors are summarized in Table 1. Almost 70% of tumors in our series were benign, with three-quarters of tumors in this group being pleomorphic adenomas. In both groups, most tumors were of salivary origin, with the remaining histological subtypes made up of diverse soft-tissue and neurogenic tumors. The neurogenic tumors originated from the 10th cranial nerve (CN X) in two cases and one case each from the CN VII, CN IX and CN XII. In the two remaining patients, the nerve originating the tumor could not be identified.

### Pre-operative management, surgical techniques, postoperative complications and adjuvant treatment (Table 2)

**Table 2:** Management

Feature	No. of cases	%
Surgery		
Pre-operative embolization		
*No*	42	87.5
*Yes*	6	12.5
Approach		
*Transcervico-parotid with partial/subtotal/total parotidectomy*	28	58.3
*Transoral only*	7	14.6
*Transcervical only*	4	8.3
*Transcervico-parotid with mandibular split and without parotidectomy*	3	6.3
*Transcervico-parotid with mandibular split and with subtotal parotidectomy*	1	2.1
*Combined transcervical and transoral*	3	6.3
*Combined intra- and extracranial*	1	2.1
Tracheotomy		
*No*	40	83.3
*Yes*	8	16.7
Neck dissection		
*No*	40	83.3
*Yes*	8	16.7
Non-surgical		
Adjuvant therapy		
*No adjuvant therapy*	33	68.8
*Radiotherapy*	14	29.1
*Chemotherapy*	1	2.1

**Fig. 2:**
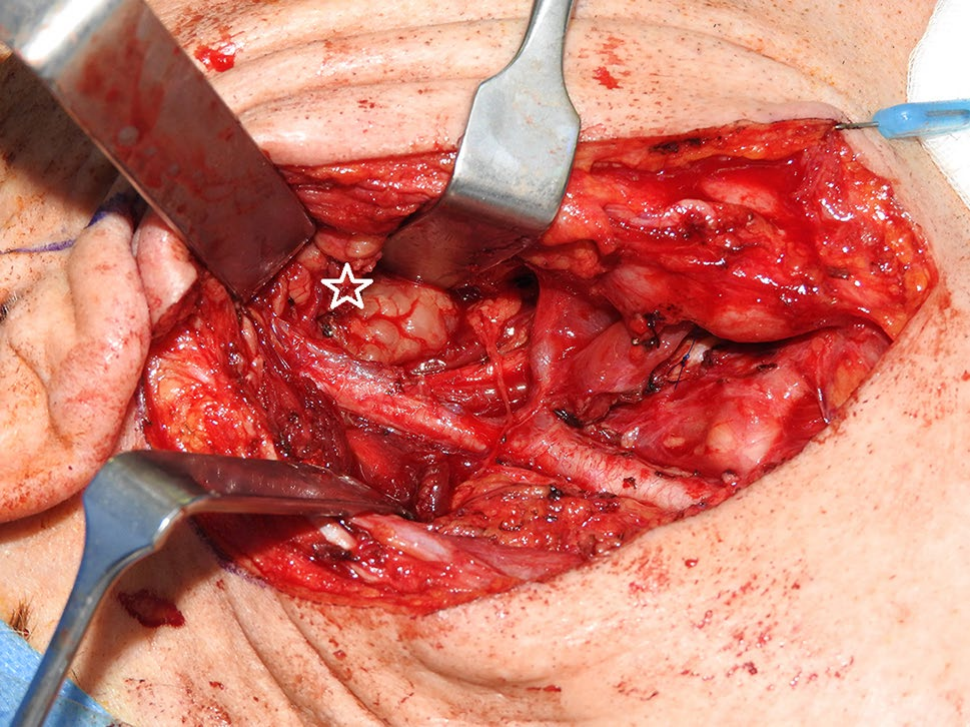
Transcervico-(parotid) approach of a parapharyngeal pleomorphic adenoma (star)

All patients except one underwent surgical removal of primary tumors. Pre-operative embolization occurred in six patients (12.5%), which exhibited radiological signs of increased blood supply to the neoplasm with feeders amenable to embolization. Exclusively external approaches were performed in 37 cases (77.1%). These approaches were mostly transcervical and through the parotid region with facial nerve identification (transcervico-parotid approach), and only in a few cases purely transcervical without identifying the facial nerve; rarely combined with mandibular split. In one patient, craniotomy was required. Tumors were amenable to exclusive transoral resection in seven cases (14.6%). Other approaches are summarized in Table 2. Tracheotomy and neck dissection were performed each in 16.7% of patients. Figure 2 shows a transcervical approach in a parapharyngeal pleomorphic adenoma.

Surgical complications were divided into minor/reversible and major/irreversible (Table 3). Temporary (resolving within 6 months) and permanent CN palsies were the most frequent postoperative complications observed. Of the 30 patients with temporary CN palsy (VII and XII), 82.1% had benign tumors and the remaining 17.9% had malignant tumors. The proportion of benign and malignant tumors in case of definitive palsy was 50%/50%. All these palsies occurred following transcervico-parotid approaches, except one case of temporary CN VII and one permanent CN XI palsy after transoral resection. Adjuvant therapy (exclusive radiotherapy in most cases and chemotherapy in a single case of undifferentiated sarcoma) was administered in approximately one-third of the patients, corresponding to the percentage of malignant tumors.

**Table 3:** Postoperative complications and tumor outcome

Feature	No. of cases	%
Minor complications (transient)		
*CN VII palsy*	28	58.3
*Dysphagia*	22	45.8
*Trismus*	9	18.8
*CN XII palsy*	2	4.2
*Horner syndrome*	1	2.1
*None*	7	14.6
Major complications (permanent)		
*CN VII palsy*	6	12.5
*CN X palsy*	5	10.4
*CN XII palsy*	2	4.2
*CN V impairement*	1	2.1
*CN XI palsy*	1	2.1
*CN IX palsy*	1	2.1
*Trismus*	1	2.1
*Conductive hearing loss*	1	2.1
*None*	32	66.7
Recurrence		
*No*	40	83.3
*Yes*	8	16.7
Status at last follow-up		
Alive without disease	41	85.4
Alive with disease	3	6.2
Dead with disease	3	6.3
Dead without disease	1	2.1

*CN* cranial nerve

### Outcome (Table 3)

The median follow-up time was 34.9 months after surgery, with a minimal follow-up of 3.5 months following by non-tumor-related death and a maximal time of over 19 years. Recurrences were diagnosed in eight patients, out of which three had benign and five malignant tumors. All of the malignant recurrences but a rhabdomyosarcoma in a 4-year-old boy underwent surgical salvage followed by adjuvant treatment (radiotherapy, systemic treatment). All benign recurrent tumors underwent surgery alone (two pleomorphic adenoma, one vagal schwannoma).

At the end of the follow-up period, only four patients were deceased (three patients with recurrent disease: one poorly differentiated salivary duct carcinoma, one rhabdomyosarcoma and one carcinoma ex pleomorphic adenoma; one patient due to cardiovascular causes shortly after the postoperative period).

## Discussion

In this series of 48 patients with primary PSNs, our main findings were as follows: (1) most neoplasms were benign and had a non-specific presentation, featuring a swelling of the parotid region/mandibular angle, dysphagia and pain; (2) salivary gland and neurogenic tumors were the most common PPS neoplasms accounting for 80% of cases, with soft-tissue sarcomas being overall rare; (3) both FNAC and transoral incisional biopsies are highly accurate to distinguish between benign and malignant neoplasms, while FNAC provides correct information on the final histopathological diagnosis in a limited number of cases; (4) the vast majority of PSNs required open surgical approaches; (5) cranial nerve palsy was the most common short- and long-term complication, with malignant tumors more commonly featuring permanent palsy; and (6) the outcome depends on the nature of the tumor, with an overall good control rate for both benign and malignant tumors.

PSNs constitute a heterogeneous group of tumors from a histological perspective. Consistent with most publications, we found a majority of benign tumors with pleomorphic adenomas and schwannomas as the most frequent individual entities [7, 13, 18]. Systematic reviews including large numbers of patients consistently show a proportion of benign tumors exceeding 75% of all cases [7, 10, 11, 19]. Despite the histological heterogeneity, an essential step in the diagnostic approach of PSNs is to determine their benign vs. malignant nature, a feature difficult to establish solely based on clinic and imaging. Imaging obviously retains important indications, especially when it comes to assessing the macroscopic tumor extension and its anatomic relationships with important vascular and nervous structures. As pointed out by Locketz et al. [19], some authors underline the important role of MRI in distinguishing between pre- and post-styloid tumors, the former being predominantly salivary (and therefore mostly benign), the latter consisting of other tumors types. Moreover, surgical approaches to the poststyloid space carries specific forms of morbidity with an important impact on quality of life, such as Horner’s syndrome, vagal palsy and first bite syndrome [19–21]. Depending on the histological subtype, further imaging such as PET/CT may be needed in the work-up to rule out neck lymph node as well as systemic metastases [19].

Cytological and/or histopathological diagnosis is of immense importance when planning the extent of resection needed for the primary tumor as well as the need for management of the neck. An accurate idea of potential malignancy allows more precise planning of the extension of the surgery and optimal pre-operative patient information.

With respect to diagnostic methods, reported rates of correct diagnosis of PSNs with FNAC range between 40 and 90% [18, 22–25]. In our study, we found US-guided FNAC to have a 100% specificity and positive predictive value to predict malignancy, but 8/29 FNACs were not diagnostic at all. Regarding correlation with definitive histopathology, FNAC properly diagnosed most pleomorphic adenomas, but was not more informative in the remaining cases. Given the substantial histological heterogeneity of salivary gland tumors, determining the exact subtype of tumor based only on FNAC is a well-known challenge [18, 26, 27]. Transoral biopsies should be performed only in cases where transoral resection is feasible and intended, because it may complicate later transcervico-parotid tumor removal and be associated with tumor spillage. Another alternative for inaccessible tumors is CT-guided core needle biopsy (CNB). CNB allows obtaining samples for histopathological analysis with a high accuracy and a low rate of complications [28, 29].

**Fig. 3:**
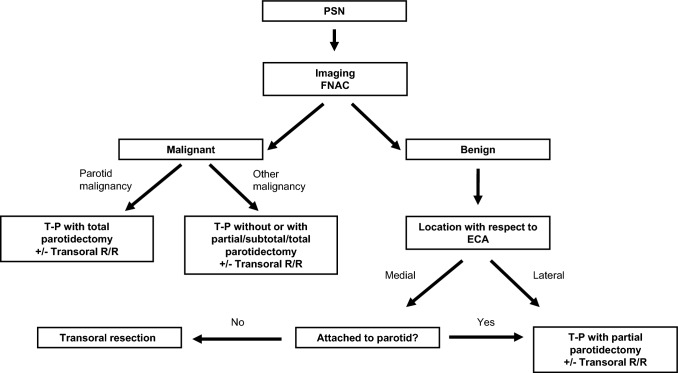
Algorithm for the selection of the appropriate surgical approach in the management of parapharyngeal space neoplasms. Pre-operative carotid artery balloon occlusion test should be performed if the internal carotid artery is radiologically infiltrated or at risk for injury during surgery. Different approaches can be combined according to tumor localization. Mandibular split should be avoided. *ECA* external carotid artery, *FNAC* fine-needle aspiration cytology, *PSN* parapharyngeal space neoplasm, *R/R* resection/release, *T-P* transcervico-parotid approach with facial nerve identification

Given the complexity of surgical approaches to the PPS and the potential for severe complications and morbidity, a standardized therapeutic approach is essential. Given the overall low incidence of PSNs, a high suspicion index is essential. Upon completion of the diagnostic work-up, in case of malignant tumor, the approaches depend on the tumor origin. Figure 3 depicts our algorithm for the selection of the appropriate surgical approach in the management of PSNs. Most approaches were transcervical and through the parotid region with facial nerve identification (transcervico-parotid approach). In case of parotid malignancies originating in the deep lobe, we perform primarily a total parotidectomy with intra-operative facial nerve identification via a transcervico-parotid approach combined with a transoral resection/release, depending on the extension. This helps often to obtain margins that are more appropriate medially. Other malignancies located in the middle and inferior portion of the PPS are resected through a transcervico-parotid approach. Depending on the extension, this resection can also be combined with a partial/subtotal/total parotidectomy and the above-mentioned transoral resection/release. In case of benign tumors located medially to the external carotid artery, an exclusive transoral resection may be attempted. In case of benign tumors located laterally to the external carotid artery, a transcervico-parotid approach with most often a partial parotidectomy without respecting large margins is chosen. An internal carotid artery occlusion test should be performed if any infiltration is suspected or if there is a possibility of its injury or sacrifice, even before transoral resection of benign lesions with limited exposure and lack of control of the great neck vessels [11, 12]. A very helpful surgical step for better exposure (> 50%) is prognathic dislocation of the mandible, following stylomandibular ligament and stylohyoid muscle division [30]. Mandibular split should be avoided and if indicated, the inferior alveolar nerve must be preserved [12].

Pre-operative embolization is helpful if the tumor is suspicious for hemangioma or exhibits increased vascularization [7, 13, 30–33]. In our study, this was the case in six patients (12.5%). However, the definitive histopathological result of these cases revealed only one hemangioma.

All but seven patients required open cervical approaches to the PPS. In contrast with many other surgical progresses, the open approach for PSN removal is still the state of the art. Transcervico-parotid approach allows better surgical exposure and results in less frequent damage to the neural structures according to the literature [7, 13, 30–33]. A temporary mandibular split may contribute to better access to the PPS and is necessary in not more than 10% of the cases, which is consistent with our findings (8.4%) [6, 7, 12–14, 30]. Nonetheless, small benign and avascular neoplasms with a predominantly luminal oropharyngeal extension and medial to the external carotid artery may be managed by transoral resection with excellent functional and cosmetic results [7, 9, 12, 13, 32, 34]. In recent articles, successful transoral robotic surgery (TORS) for PSNs tumors has been described [34–36]. According to the authors, a FNAC with a “benign” result and pre-operative image indicating no tumor infiltration of important cranial structures can make TORS suitable in individual cases. However, the authors also state that the lack of tactile feedback in this challenging anatomical region may be a major disadvantage over the typical open surgical approach [34, 35].

The most frequent complications previously reported in the literature are consistent with our results and include lesions of variable degrees to the CNs contained within the PPS (i.e., CNs VII, IX, X and XII). Neurogenic and malignant lesions have a greater risk of CN, as was the case in our cohort [2, 7, 9, 12]. According to the literature, injury to the vagal nerve is the most common complication, affecting up to 13% of all patients [7–9]. In our series, the most frequent complications were CN X and VII injury with a permanent palsy in 10.4% and 12.5% of the patients, respectively. Close follow-up should be considered to assess swallowing and speech difficulties, especially if more than one CN is involved.

Rates of recurrences depend on the tumor type [7, 9, 33, 35, 37]. The systematic review of Kuet et al. [9] reported an average 5-years progression-free survival rate of 93% for benign and 61% for malignant diseases. In our series, local recurrence was observed in two patients (7.4%) with benign and in four patients (30.8%) with malignant tumors. Recurrence of malignant tumors occurred on an average 6.4 months after primary therapy. Thus, all PSN types (benign and malignant) should benefit from clinical and radiological follow-up to assess recurrence after therapy. Such follow-up should be tailored to the tumor type.

This study is limited by its retrospective design and the rare incidence of this entity results in smaller subgroups. During the last two decades, different surgeons with different levels of experiences performed the resections, making it difficult to compare the postoperative findings/complications. In addition, the treatment modalities and pre-operative imaging changed during the last 20 years. We can perform today pre-operative imaging with higher resolution (e.g. PET-CT scan), making it easier to plan the extent of surgery. More precise radiotherapy and more compatible chemotherapy were added to the changes of treatment modalities. All these mentioned aspects make it difficult to compare our subgroups and can be seen as a weakness of our study.

## Conclusions

We present one of the largest recent studies on PPS tumors treated in one center. Given the low incidence of these tumors, our results contribute to the existing sparse evidence regarding the management and outcome of such tumors.

Neoplasms of the PPS are rare and most often present with swelling of the parotid gland and the region under the mandible angle. Consistent with the literature, two thirds of patients in our study had benign tumors, and pleomorphic adenoma and schwannoma were the most frequent entities. FNAC and/or transoral biopsy in combination with radiographic imaging are helpful in many cases to plan the extent of primary surgery.

Good exposure of the surgical site is necessary to achieve complete resection and to minimize trauma to the nerves and main vessels contained in the PPS. Therefore, open surgery is still the state of the art. In selected small benign lesions, medially to the external carotid artery, transoral resection may be indicated.

The most frequent per-/post-operative complications include temporary and/or permanent CN palsy of the nerves contained in the PPS. Neurogenic and malignant lesions have a greater risk of CN injury. Surgeons must inform their patients pre-operatively about potential outcomes. All patients with malignant tumors received adjuvant therapy (radiotherapy). The recurrence rate in malignant PSNs is high. PPS tumors should be followed-up closely to assess recurrence as early as possible.

## Data Availability

Available via corresponding author.

## Material availability

Available via corresponding author.

## Code availability (software application or custom code)

Available via corresponding author.
